# ﻿Description and phylogenetic analysis of two new *Episinus* (Araneae, Theridiidae) species from China

**DOI:** 10.3897/zookeys.1125.90212

**Published:** 2022-10-19

**Authors:** Fengjie Liu, Ingi Agnarsson, Jie Liu, Yang Zhu

**Affiliations:** 1 Hubeiate Key Laboratory of Regional Development and Environmental Response, Faculty of Resources and Environmental Science, Hubei University, Wuhan 430062, China; 2 State Key Laboratory of Biocatalysis and Enzyme Engineering, School of Life Sciences, Hubei University, Wuhan 430062, Hubei, China; 3 Faculty of Life and Environmental Sciences, University of Iceland, Sturlugata 7, 102 Reykjavik, Iceland; 4 School of Nuclear Technology and Chemistry and Biology, Hubei University of Science and Technology, Xianning 437100, Hubei, China; 5 Wuhan Lvjia Technology Co., Ltd, Wuhan 430114, Hubei, China

**Keywords:** New species, phylogenetic, taxonomy

## Abstract

The spider genus *Episinus* Walckenaer, 1809 currently contains 66 species worldwide, mostly in warm temperate to tropical areas. This paper describes two new Chinese *Episinus* species: *E.ornithorrhynchus***sp. nov.** (♂♀) and *E.papilionaceous***sp. nov.** (♀). We add these two new and one known *Episinus* species to the phylogenetic data matrix of Liu et al. 2016 and reanalyze the data. The new phylogeny recovers the monophyly of *Episinus* and supports its division into two groups, a finding also supported by morphology.

## ﻿Introduction

Currently, the Theridiidae Sundevall, 1833, constitutes one of the largest families of spider, with 2539 described species in 125 genera distributed worldwide. In China, there are 380 species of Theridiidae, and they belong to 54 genera (World Spider Catalog 2022). The theridiid subfamily Spintharinae currently consists of 10 genera: *Brunepisinus* Yoshida & Koh, 2011; *Chrosiothes* Simon, 1894; *Episinus* Walckenaer in Latreille, 1809; *Moneta* O. Pickard-Cambridge, 1870; *Pycnoepisinus* Wunderlich, 2008; *Spintharus* Hentz, 1850; *Thwaitesia* O. Pickard-Cambridge, 1881; *Stemmops* O. Pickard-Cambridge, 1894; *Neopisinus* Marques, Buckup & Rodrigues, 2011; *Janula* Strand, 1932 (Agnarsson 2004; Arnedo et al. 2004; Marques et al. 2011; Durán-Barron et al. 2013; Liu et al. 2016; Vanuytven 2021; Rodrigues et al. 2022).

At present, the genus *Episinus* has 66 described species and of which only 10 have been reported in China (World Spider Catalog 2022). More than half of these species are widespread and also recorded in the Nearctic, Japan, Europe, and Africa (Levi 1955; Yoshida 1985; Okuma 1994; Zhu 1998; Song et al. 1999). *Episinus* differs from other genera of the Spintharinae by the straight shape of the opisthosoma in lateral view, and in dorsal view with median or posterior humps (Durán-Barron et al. 2013; Rodrigues et al. 2022). Rodrigues et al. 2022 provided a phylogenetic framework based on a morphological analysis, which suggested that *Janula* and some species of *Episinus* belong to the same group, and the genus *Episinus* appeared polyphyletic. The genus *Moneta* is similar to *Episinus*, but according to Okuma (1994) can be separated by the alignment of the eyes, the length ratio of the metatarsus to the tarsus, and the structure of the male palp. Our phylogenetic results clearly differentiate between these genera and strongly support the monophyly of the genus *Episinus* (Fig. 6).

We describe herein two new *Episinus* species from China, *E.ornithorrhynchus* sp. nov. and *E.papilionaceous* sp. nov. We add these species, as well as *E.nubilus* Yaginuma, 1960, to the data matrix of Liu et al. (2016) and rerun phylogenetic analyses.

## ﻿Materials and methods

All specimens were kept in absolute ethanol and examined with an Olympus SZX7 stereomicroscope; details were further investigated with an Olympus BX51 compound microscope. Male palps and female genitalia were examined and photographed after dissection from the spider bodies, epigynes were cleared with Proteinase K, and palps were studied after immersion in KOH; habitus photos were obtained using a Leica 205C digital microscope. Left palps are illustrated. All specimens are deposited at the Centre for Behavioural Ecology and Evolution, College of Life Sciences, Hubei University, Wuhan, China (CBEE).

Leg measurements are shown as total length (femur, patella, tibia, metatarsus, tarsus). The number of spines is listed for each segment in the following order: prolateral, dorsal, retrolateral, and ventral (in femora and patellae ventral spines are absent, and the fourth digit is omitted in the spination formula). The terminology used in the text, figure legends, and palp homologies follow Agnarsson (2004) and Agnarsson et al. (2007). All measurements are given in millimeters.

### ﻿Molecular data

We used the dataset from Liu et al. (2016) and added to it *E.ornithorrhynchus* sp. nov. and *E.papilionaceous* sp. nov., as well as *E.nubilus* Yaginuma, 1960. The final dataset includes 62 theridiid genera and two outgroups representing Nesticidae and Synotaxidae. Sequences for two mitochondrial genes: cytochrome c oxidase subunit I (COI) and ribosomal RNA16S (16S), and three nuclear genes, ribosomal RNAs 18S (18S) and 28S (28S) and histone (H3), were collected for the newly added species. The phylogenetic data collection and analytical methods used conform to Liu et al. (2016).

The appropriate models for the Bayesian analysis were selected with jModelTest2 on XSEDE (2.1.6) (Darriba et al. 2012) using the Akaike information criterion (AIC) (Posada 2008). Bayesian analysis for morphology was carried out using MrBayes 3.2.7a on XSEDE (Huelsenbeck and Ronquist 2001) and maximum likelihood with IQ-Tree stable release 1.6.12 (Chernomor et al. 2016; Nguyen et al. 2015) of individual gene trees as well as concatenated matrices. All large analyses were run in parallel on the CIPRES cluster at the San Diego Supercomputing Center (Miller et al. 2010).

### ﻿Abbreviations used

**ALE**—anterior lateral eyes, **AME**—anterior median eyes, **Atr**—atrium, **C**—conductor, CD—copulatory duct, **CY**—cymbium, **E**—embolus, **FD**—fertilization duct, **MA**—median apophysis, **MS**—medium septum, **PLE**—posterior lateral eyes, **PME**—posterior median eyes, **I–IV**—1^st^ to 4^th^ leg, **S**—spermathecae, **Teg**—tegulum, **CBEE**—Centre for Behavioural Ecology and Evolution, College of Life Sciences, Hubei University, Wuhan, China.

## ﻿Results

### ﻿Taxonomy

**Family Theridiidae Sundevall, 1833**

#### ﻿Genus Episinus

Taxon classificationAnimaliaAraneaeTheridiidae

Walckenaer in Latreille, 1809

35F15487-B106-5344-BE6B-86A47C35EB06

##### Type species.

*Episinustruncates* Latreille, 1809.

#### ﻿ Episinus ornithorrhynchus sp. nov.

Taxon classificationAnimaliaAraneaeTheridiidae

243E11C8-179A-5B44-86D2-DB38C8F5637A

https://zoobank.org/D15231DB-9F9B-4A5D-A517-40A941EA92B6

**Figure 1.**
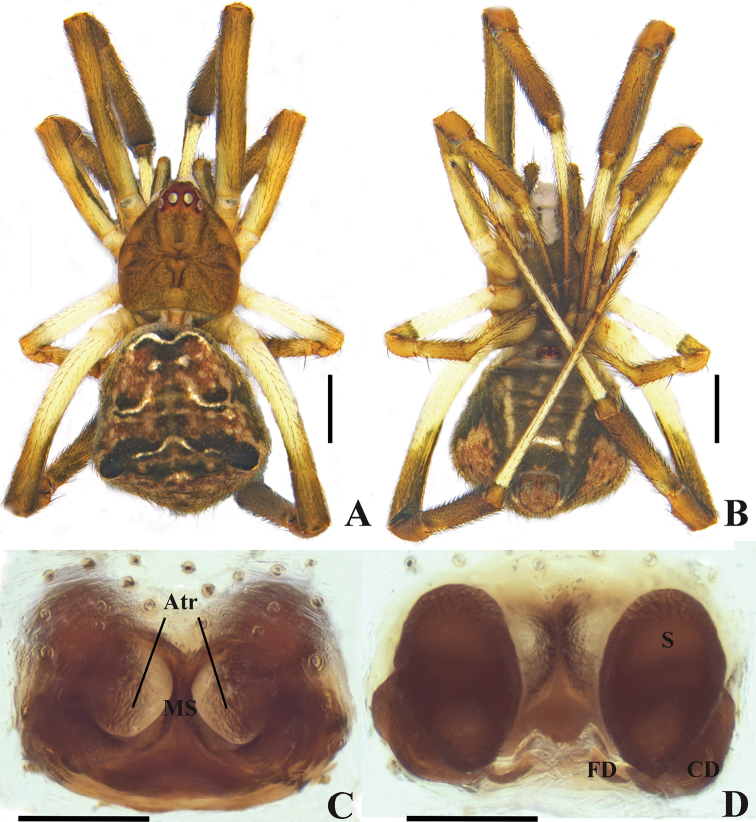
*Episinusornithorrhynchus* sp. nov. **A, B** female habitus **A** dorsal **B** ventral **C, D** epigynum in alcohol **C** ventral **D** dorsal. Abbreviations: Atr—atrium, CD—copulatory duct, FD—fertilization duct, MS—medium septum, S—spermathecae. Scale bars: 1 mm (**A, B**); 0.1 mm (**C, D**).

**Figure 2.**
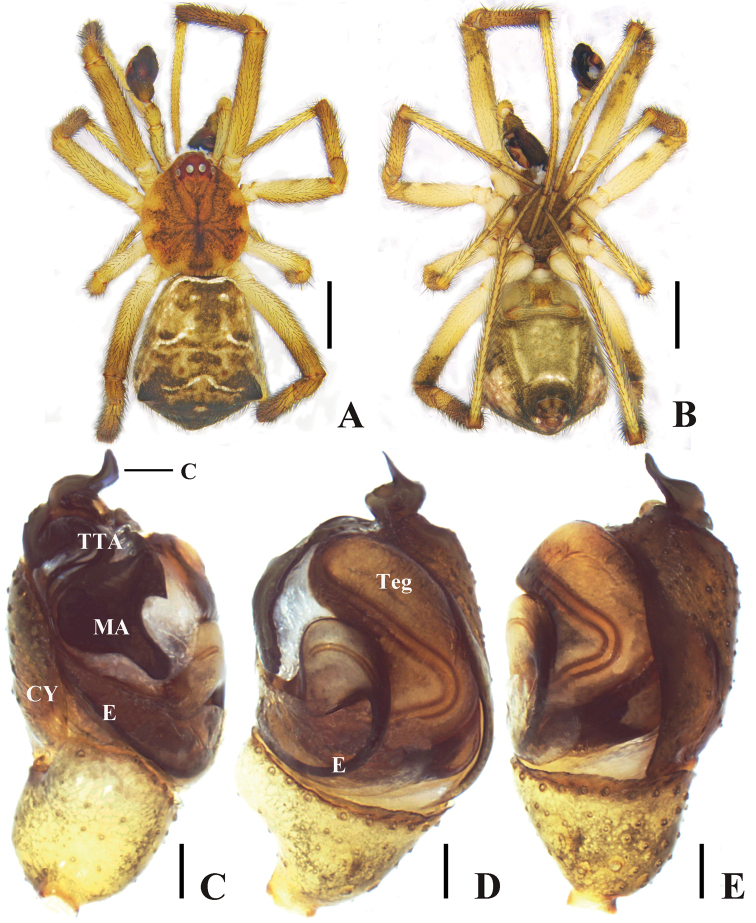
*Episinusornithorrhynchus* sp. nov. **A, B** male habitus **A** dorsal **B** ventral **C–E** palp in alcohol **C** prolateral **D** ventral **E** retrolateral. Abbreviations: C—conductor, CY—cymbium, E—embolus, MA—Median apophysis, Teg—tegulum. Scale bars: 1 mm (**A, B**); 0.1 mm (**C–E**).

**Figure 3.**
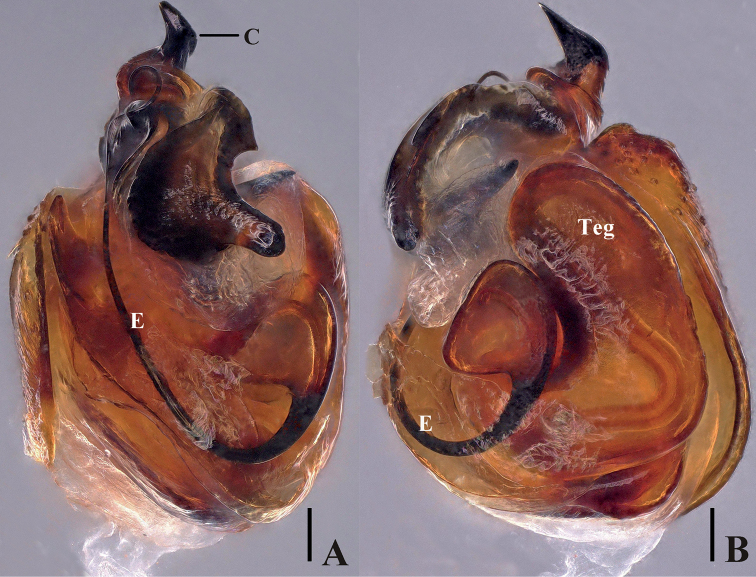
*Episinusornithorrhynchus* sp. nov. **A, B** male palp (previously soaked with KOH) in alcohol. **A** prolateral **B** ventral. Abbreviations: C—conductor, E—embolus, Teg—tegulum. Scale bars: 0.1 mm.

Figs 1A–D , 2A–E , 3A, B , 5

##### Type material.

***Holotype***: ♂, China, **Yunnan Province**: Mengsong Town, Mengsong Township Central Primary School, (22°4'12"N, 100°33'36"E, 1340 m alt.), 1 August 2020, Z.C. Li, R. Zhong, W.Z. Deng, W. Zhang, and Y.T. Zhang leg. ***Paratypes***: 1♂2♀, same data as holotype; **Yunnan Province**: 2♀, Menglun Town, Baka Xiaozhai, (22°4'12"N, 101°12'0"E, 810 m alt.), 24 July 2020, Z.C. Li, R. Zhong, W.Z. Deng, W. Zhang, and Y.T. Zhang leg.

##### Diagnosis.

Males are similar to *E.baoshanensis*Liu et al. (2019) but can be distinguished from them by the palpal structure: 1) embolus extends along the left lower lateral aspect of the genital bulb to the posterior margins, but the embolus mainly encircles the left upper lateral and anterior margins of the bulb in *E.baoshanensis* (Figs 2C, D, 3A, B); 2) conductor with a sharp tip in ventral view and not bifurcated, but conductor sclerotized and tip bifurcated in *E.baoshanensis* (Figs 2C, 3A). Females are similar to *E.nubilus* but can be distinguished from them by the direction of the copulatory duct: fertilization ducts are slightly shorter in the new species, but long and C-shaped in *E.nubilus* (Fig. 1D).

##### Etymology.

The specific name is derived from the Latin adjective *ornithorrhynchus*, meaning bird’s beak, referring to the shape of the conductor; adjective.

##### Description.

**Male** (paratype): total length 3.79; prosoma length 1.60, width 1.39; opisthosoma length 2.19, width 1.67; eye diameters: ALE 0.10, AME 0.10, PLE 0.10, PME 0.10; eye interdistances: AME–AME 0.10, AME–ALE 0.05, PME–PME 0.10, PME–PLE 0.05; clypeus height 0.24; leg measurements: I 6.84 (1.83, 0.41, 2.11, 1.78, 0.71), II 5.04 (1.46, 0.39, 1.14, 1.35, 0.70), III 3.72 (1.11, 0.35, 0.73, 0.96, 0.57), IV 7.76 (2.28, 0.52, 1.70, 2.40, 0.86). Leg formula: IV, I, II, III. Overall, the color was slightly lighter and as in females (Fig. 2A, B).

Palp (Figs 2C–E, 3A, B). Subtegulum mostly covered by tegulum. Embolus originates at center of palp, a slightly triangular basement that curves upward clockwise. Conductor sclerotized, with a sharp tip extending over apex of cymbium.

Female (holotype): total length 4.25; prosoma length 1.70, width 1.49; opisthosoma length 2.55, width 2.05; eye diameters: ALE 0.10, AME 0.10, PLE 0.10, PME 0.10; eyes interdistances: AME–AME 0.10, AME–ALE 0.06, PME–PME 0.08, PME–PLE 0.08; clypeus height 0.25; leg measurements: I 7.44 (2.05, 0.61, 1.79, 2.23, 0.76), II 5.06 (1.52, 0.50, 1.06, 1.30, 0.68), III 3.91 (1.28, 0.33, 0.68, 0.93, 0.69), IV 8.25 (2.36, 0.80, 1.65, 2.46, 0.98). Leg formula: IV, I, II, III. Carapace yellowish to reddish brown, with a deep transverse depression. Sternum gray-black, slightly longer than wide. Femur and tibia to tarsus yellowish white, and distal patella reddish brown (Fig. 1A, B). Opisthosoma yellowish to orange-brown, with longitudinal white stripes in ventral view, extending posteriorly and short humps on each side. Opisthosoma dorsally grayish black, with a rectangular dark spot (Fig. 1B).

Epigyna (Fig. 1C–D). Atria separated by median septum. Spermathecae slightly oval with two parallel stripes on middle part; fertilization ducts shorter.

##### Variation.

Total length male 3.75–3.79 (*n* = 2), female 4.10–4.25 (*n* = 4).

##### Distribution.

China (Yunnan Province) (Fig. 5).

#### ﻿ Episinus papilionaceous sp. nov.

Taxon classificationAnimaliaAraneaeTheridiidae

D80E3432-EC01-51E3-85F0-E2353640074A

https://zoobank.org/83AD0CDA-5904-4EC9-8BB7-5E14133D2CC6

Figs 4A–D , 5

##### Type material.

***Holotype***: ♀, CHINA, **Hunan Province**: Zhangjiajie City, Badagong Mountain National Nature Reserve (29°47'24"N, 110°6'0"E, 1395 m alt.), 2 June 2018, F.X. Liu and Z.C. Li leg.

##### Diagnosis.

**Figure 4.**
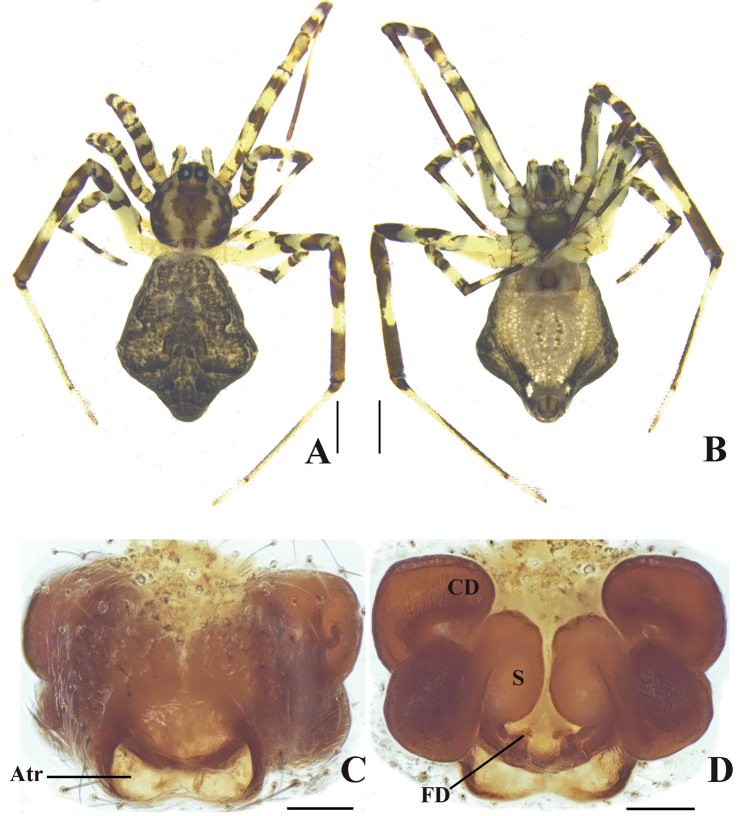
*Episinuspapilionaceous* sp. nov. **A, B** female habitus **A** dorsal **B** ventral **C, D** epigynum in alcohol **C** ventral **D** dorsal. Abbreviations: Atr—atrium, CD—copulatory duct, FD—fertilization duct, S—spermathecae. Scale bars: 1 mm (**A, B**); 0.1 mm (**C, D**).

This new species is similar to *E.xiushanicus* Zhu, 1998 in having a peach-shaped structure at the posterior part of the epigynal field but can be distinguished from them by the following characteristics: 1) abdomen without spinous process in ventral view, but the ventral protuberance is spinous in *E.xiushanicus* (Fig. 4A); 2) spermathecae slightly S-shaped in the new species but W-shaped in *E.xiushanicus* (Fig. 4D).

##### Etymology.

The specific name is derived from the Latin adjective *papilionaceous*, meaning butterfly-shaped, referring to the shape of the epigynum; adjective.

##### Description.

**Male** unknown. **Female** (holotype): total length 4.29. Prosoma length 1.55, width 1.44; opisthosoma length 2.74, width 2.55; eye diameters: ALE 0.11, AME 0.11, PLE 0.09, PME 0.09; eye interdistances: AME–AME 0.10, AME–ALE 0.05, PME–PME 0.15, PME–PLE 0.08; clypeus height 0.50; leg measurements: I 8.00 (2.48, 0.61, 1.83, 2.47, 0.61), II 5.46 (1.78, 0.41, 1.17, 1.42, 0.68), III 4.77 (1.43, 0.34, 0.76, 1.83, 0.41), IV 8.57 (2.75, 0.69, 2.09, 2.34, 0.70). Leg formula: IV, I, II, III. Carapace yellowish brown, with two parallel elongate yellowish-white markings posterior to eyes, and submarginal black markings laterally. Posterior median eyes slightly mound-shaped and surrounding area black. Sternum grayish yellow, slightly longer than wide. Legs yellow, with brown markings. Opisthosoma yellowish black, with irregular black stripes in ventral view and extending posteriorly. Opisthosoma dorsally yellowish black, with tiny white to black dots (Fig. 4A, B).

Epigyna (Fig. 4C, D). Ventral view wider than long, with a peach-shaped structure at posterior margin. Copulatory ducts thick and curved. Fertilization ducts arising medially.

##### Distribution.

**Figure 5.**
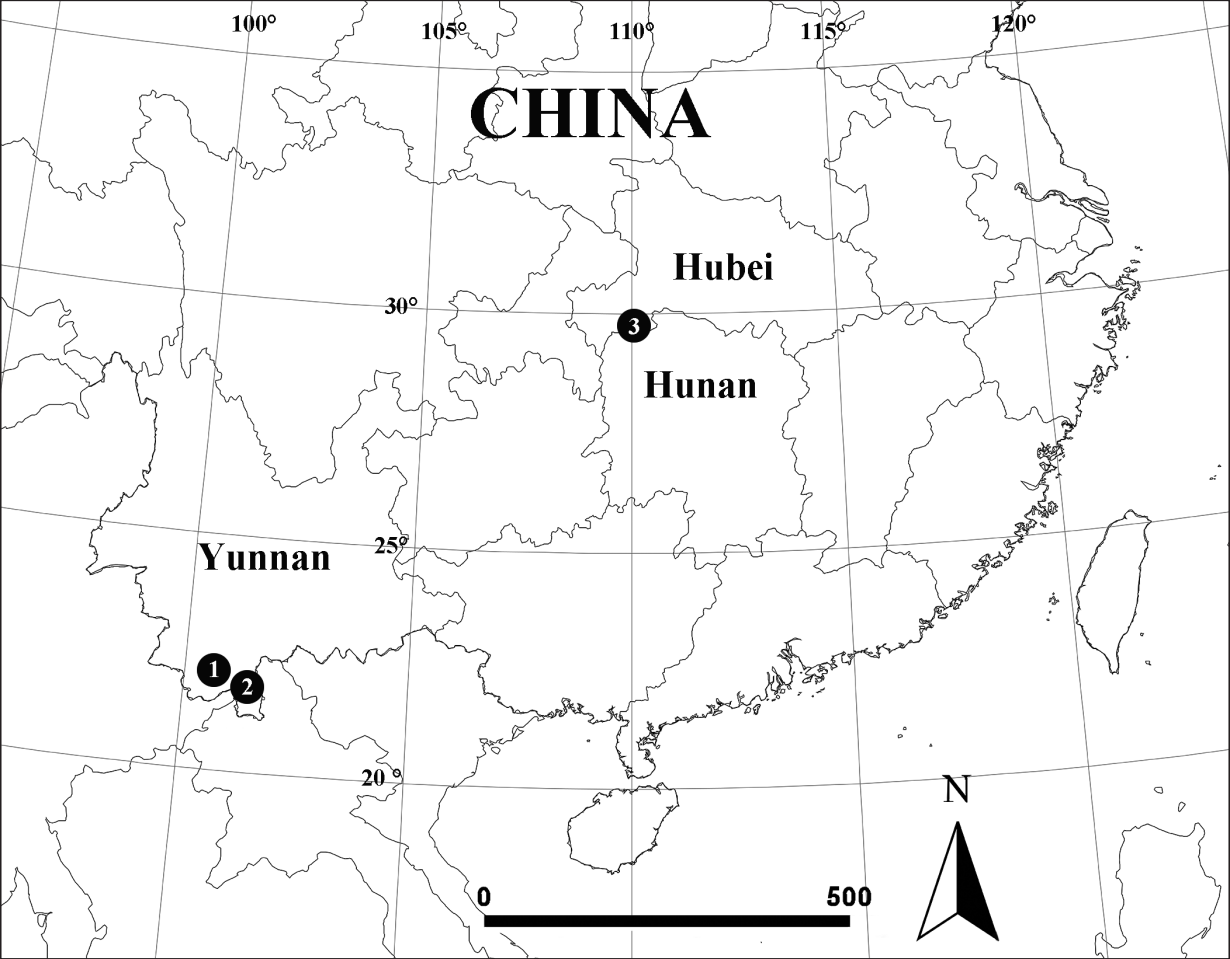
Locality records for two species of *Episinus*: 1 = *Episinusornithorrhynchus* sp. nov., 2 = *Episinuspapilionaceous* sp. nov.

China (Hunan Province) (Fig. 5).

### ﻿Phylogenetic analyses

Our phylogeny tree supports the fundamental findings of prior studies, the monophyly of Theridiidae and seven subfamilies of Theridiidae: Latrodectinae, Pholcommatinae, Argyrodinae, Hadrotarsinae, Spintharinae, Anelosiminae, and Theridiinae.

**Figure 6.**
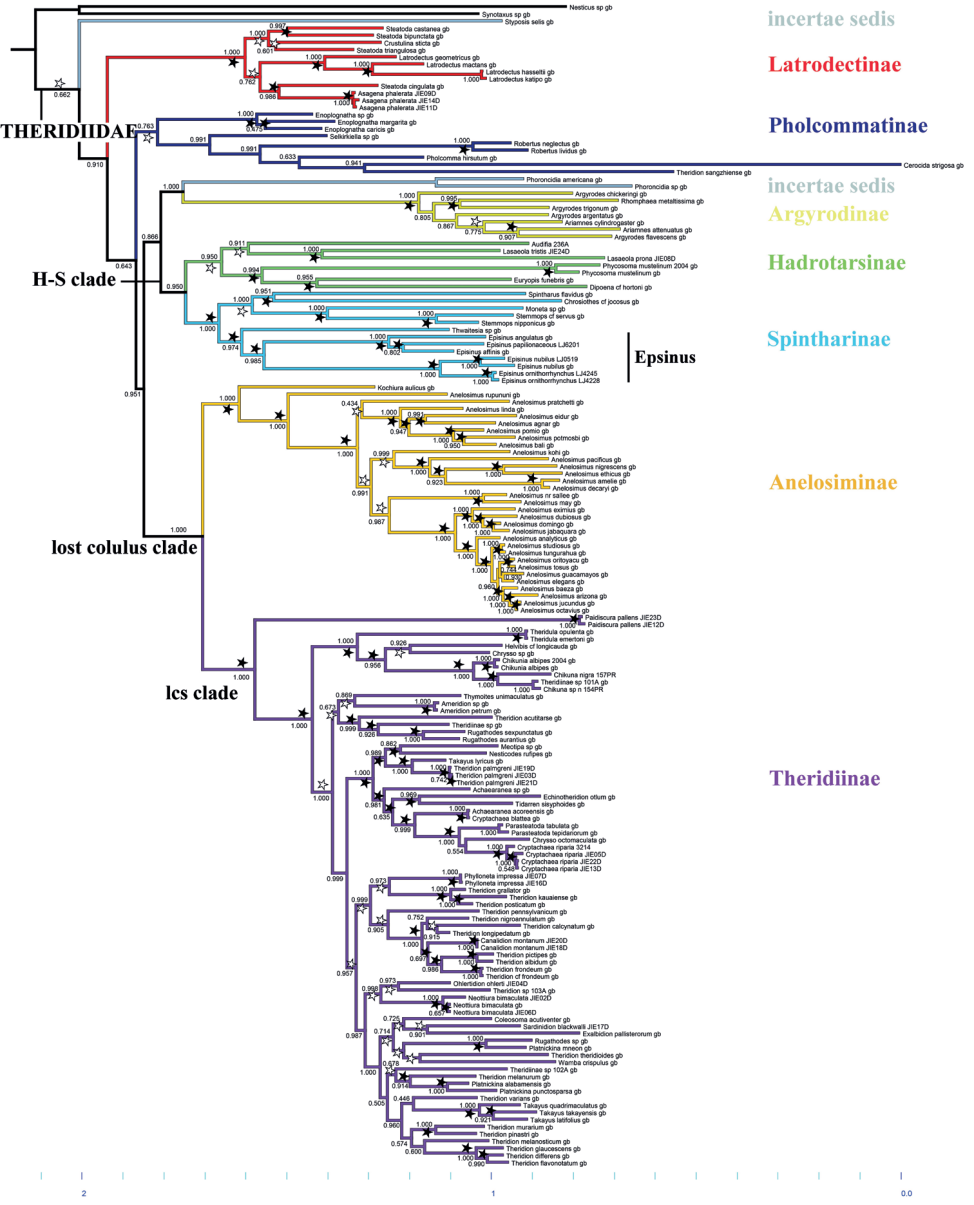
Bayesian analysis of molecular and morphological data of the focal dataset, a matrix excluding all taxa with over 70% missing data. Numbers on nodes are posterior probabilities; bootstrap support from ML analyses is indicated: solid stars indicate bootstrap support values >95%, the gray stars > 50–95%, and nodes with less than 50% support lack stars.

From the results (Fig. 6), the Latrodectinae is the earliest branching subfamily. Pholcommatinae is polyphyletic and redefined by removing *Styposis* and *Phoroncidia* that must be placed in insertae sedis until further data become available. Argyrodinae is in the same monophyletic group as the *Phoroncidia*, and these are sisters to Spintharinae and Hadrotarsinae. Our results strongly support the sister relationship between Spintharinae and Hadrotarsinae, two specialized groups with most of their species either building reduced webs or having abandoned web building and hunting ants as prey. Anelosiminae is recovered as a monophyletic sister to Theridiinae.

Our results strongly support the monophyly of the subfamily Spintharinae. We have also found compelling evidence to support the monophyly of the genus *Episinus*, although this is weak due to the lack of data for the most-related genera: *Janula*, *Neopisinus*, and *Brunepisinus*. Regardless, we found evidence for the first time for the division of the genus *Episinus* into two groups. The “angulatus” group to which *E.ornithorrhynchus* sp. nov., belongs is characterized by the male embolus, and the “nubilus” group to which *E.papilionaceous* sp. nov. belongs is characterized by female atria. These two groups can be clearly diagnosed based on the following morphological traits: in the “angulatus” group, the male embolus originates at the center of the palp and is close to the cymbium; in the “nubilus” group, female atria are separated by a medium septum. *Episinusnubilus* Yaginuma, 1960, here newly added to the phylogenetic matrix, was found to be closely related to *E.ornithorrhynchus* sp. nov. Our findings suggest that the genus *Moneta* O. Pickard-Cambridge, 1871 which is morphologically similar to *Episinus*, is unrelated to it, but instead related to the genus *Stemmops* O. Pickard-Cambridge, 1894. The genus *Thwaitesia* O. Pickard-Cambridge, 1881 was recovered as sister to *Epsisnus*, with strong support (Bayesian PP 97.4%, ML bootstrap 95%). This is similar to the morphological phylogeny of Durán-Barron et al. (2013) but in stark contrast with the morphological phylogeny of Rodrigues et al. (2022), which places *Thwaitesia* far from *Episinus* and *Moneta*. Unlike our study, which contains both molecular and morphological data, the relationship between the genera was discussed more from an evolutionary perspective.

## ﻿Discussion

Based on the combination of molecular and morphological data, our knowledge of the phylogeny of Theridiidae is rapidly growing (Agnarsson 2004; Liu et al. 2016). The phylogeny serves as a useful tool for comparative studies and as a guide to improved classification. Our phylogeny (Fig. 6) refined that of Liu et al. (2016) by focusing on the genus *Episinus*. Our results offer the strongest test of the monophyly of *Episinus* to date and indicate that the genus can be clearly divided into two groups, supported independently by molecular and morphological evidence.

Our results also have implications for the broader phylogeny of Theridiidae in as much as they differ from Liu et al. (2016). As found previously, the placement of the “orphan” pholcommatines *Styposis* and *Phoroncidia* is unstable across different analyses, with the current results indicating *Styposis* is close to Latrodectinae and *Phoroncidia* is close to Argyrodinae. Latrodectinae includes some of the largest species and of which the genus *Steatoda* Sundevall, 1833 is polyphyletic. *Theridionsangzhiense* Zhu, 1998 is here again placed within Pholcommatinae, most likely indicating an error in species identification of GenBank materials. Different from previous studies, we recovered Argyrodinae (plus *Phoroncidia*) as a sister to Spintharinae plus Hadrotarsinae. If this group is true, these taxa may be united by extreme reduction or loss of webs. Importantly, we recovered *Audifia* as a member of Hadrotarsinae, as expected based on morphology, but in contrast to Liu et al. (2016). This result indicates the monophyly of Hadrotarsinae, although this subfamily has strong morphological diversity (including two pairs of spermathecae and modifications of spinnerets and palpal claws). The undisputed sister relationship of Anelosiminae and Theridiinae was again supported.

Our study focuses mainly on the subfamily Spintharinae and confirmed its monophyly. The genera *Spintharus*, *Thwaitesia*, and *Episinus* all tend to build an H-shaped web with the animal facing downwards and holding onto parts of the web with its feet, presumably specializing on pedestrian prey such as ants (Agnarsson 2004; Eberhard et al. 2008). A close relationship between *Thwaitesia* and *Epsisnus* was proposed by Durán-Barron et al. (2013) based on morphology. However, also based on morphology, Rodrigues et al. (2022), found that *Thwaitesia* is unrelated to *Episinus*, and morphological evidence showed that humps are present on the abdomen in *Episinus* but absent in *Thwaitesia*. Further evidence for the relationship between *Thwaitesia* and *Episinus* may come from the structure of the egg sac (Eberhard et al. 2008). Most theridiid egg sacs have a densely spun outermost layer, while the outermost fibers are loosely woven in *Episinus* and *Thwaitesia* (Agnarsson 2004). However, there are insufficient data available on egg sacs of other spintharines.

We found support for the monophyly of *Episinus* and its division into two morphologically well-defined branches. The “angulatus” group includes *E.angulatus* (Blackwall, 1836), *E.papilionaceous* sp. nov., and *E.affinis* Bösenberg & Strand, 1906. This group differs from the “nubilus” group, which includes *E.nubilus* Yaginuma, 1960 and *E.ornithorrhynchus* sp. nov., in the origin of the male embolus and the ventral view of female epigynal. The “angulatus” group can be characterized by the male embolus originating closer to the cymbium at the lateral edge of the palp, whereas in the “nubilus” group the embolus arises in clockwise direction upward bending in the middle of the palp. The “angulatus” group presents only one large atrium in the ventral view of the female epigynal, but the “nubilus” group has two atria separated by the medium septum. The medium septum is a projection in the middle of the atrium and has a guiding function (Yin et al. 2012). In future studies of *Episinus* and Spintharinae, it will be critical to include other spintharine genera, especially those previously included in *Episinus*, such as *Janula*, *Neopisinus*, and *Brunepisinus*.

## Supplementary Material

XML Treatment for Episinus

XML Treatment for Episinus ornithorrhynchus

XML Treatment for Episinus papilionaceous

## Supplementary materials

Supplementary material 1 Table S1 Data type Genomic. Explanation note Phylogenetic analysis of the spider genus *Episinus* (Araneae: Theridiidae) and description of the two new species from China. https://binary.pensoft.net/file/759550This dataset is made available under the Open Database License (http://opendatacommons.org/licenses/odbl/1.0/). The Open Database License (ODbL) is a license agreement intended to allow users to freely share, modify, and use this Dataset while maintaining this same freedom for others, provided that the original source and author(s) are credited.Fengjie Liu, Ingi Agnarsson, Jie Liu, Yang Zhu
